# From Eardrum to Cranium: Pneumocephalus Secondary to Acute Otitis Media

**DOI:** 10.7759/cureus.43756

**Published:** 2023-08-19

**Authors:** Waseem K Naboulsi, Rebekah Lantz, Asif Khan

**Affiliations:** 1 Internal Medicine, Wright State University Boonshoft School of Medicine, Dayton, USA; 2 Internal Medicine, Miami Valley Hospital, Dayton, USA; 3 Infectious Diseases, Dartmouth Hitchcock Medical Center, Dartmouth, USA

**Keywords:** streptococcus pneumoniae, cranial air, ball-valve mechanism, inverted soda bottle effect, infectious disease pathology, empyema, infectious pneumocephalus, traumatic pneumocephalus, iatrogenic pneumocephalus, pneumocephalus

## Abstract

Pneumocephalus, or air entrapment within the cranium, is a rare but dangerous condition with a variety of causes, including trauma, surgery, or predisposing infection. Trauma is the most common etiology, as fractures provide easy access for air to become entrapped in the cranium. However, access such as via the central nervous system with leak exists. Though not as common as traumatic pneumocephalus, pneumocephalus secondary to infection is a dangerous condition. The literature is sparse on this example of ear-nose-throat infection, making it difficult to form and ascertain guidelines for the management of infectious pneumocephalus and its complications.

A 58-year-old man with a history of hypertension, obesity, type 2 diabetes mellitus, and obstructive sleep apnea who presented with a complicated case of acute otitis media associated with subdural empyema, pneumocephalus, and group A Streptococcus is presented in this case report. We describe the course of his hospital stay, management, and current infectious disease guidelines. Given the paucity of cases of infectious pneumocephalus secondary to otitis media, we aim to provide further representation for this important illness as well as encourage the use of a multimodal team of providers. In our case, it was necessary to involve the ear-nose-throat specialist as well as infectious disease and neurocritical care services.

## Introduction

Pneumocephalus, the presence of air in the cranial vault, is a rare condition with serious and potentially life-threatening consequences. It is classified into two main types: traumatic pneumocephalus, from external injuries and surgeries, and non-traumatic pneumocephalus, including infectious, iatrogenic, and spontaneous causes.

Traumatic pneumocephalus is commonly caused by skull fractures and severe head trauma or secondary to invasive neurosurgical procedures such as craniotomies. In such cases, the pneumocephalus results from communication with the external environment and cranial cavity. It is recognized that neurosurgical interventions can inadvertently introduce air through dural tears and air emboli [1,2]. Non-traumatic pneumocephalus occurs in the absence of external trauma and can occur secondary to a condition such as pneumothorax, barotrauma, or as a rare complication of sinus or middle/inner ear infections. Examples of iatrogenic causes include a complication of lumbar puncture, endoscopic shunts, or ventriculoperitoneal shunt placement [1,2].

The above etiologies may culminate in tension pneumocephalus, an air buildup that can enlarge and cause mass effects. If severe enough, patients can exhibit symptoms or even prior to symptoms develop overt seizures and possibly death [3]. Three general pathophysiologic mechanisms have been proposed. First, a ball-valve mechanism, where a one-way defect allows air to enter but not exit the cranial cavity. Second, an inverted soda-bottle effect, where cerebrospinal fluid (CSF) leak creates negative pressure for air to enter the cranial cavity. Third, the rarest cause is gas-producing infections [4]. The clinical symptoms of pneumocephalus vary by location and severity but include a spectrum of neurologic deficits secondary to mass effect, including headaches of varying severity, altered mental status, signs of increased intracranial pressure, and seizures. Confirmatory testing can be accomplished through computed tomography or magnetic resonance imaging (MRI) to determine size and location [5].

Treatment of pneumocephalus varies based on the etiology, but generally, patients are managed expectantly with 100% O2 followed by surgical intervention when a fracture or declining clinical status is present. Emergent decompression is indicated via the use of burr holes and needle aspiration [6]. When infection is suspected, treatment with a six-week course of IV antibiotics is helpful to cover for the most common causes of meningitis and should include *Streptococcus pneumoniae* coverage [7].

## Case presentation

A 58-year-old Caucasian man with a history of hypertension, non-insulin-dependent type 2 diabetes mellitus, obstructive sleep apnea, and obesity presented to the urgent care in January 2023 complaining of upper respiratory symptoms for two weeks along with worsening sinus pressure, sore throat, congestion, and otalgia over the past three days. His exam revealed bilateral ear tenderness and was otherwise neurologically normal. An exam showed middle ear effusions with intact but erythematous and bulging tympanic membranes. He was diagnosed with acute otitis media (AOM) and treated with a dose of IV empiric piperacillin/tazobactam, ketorolac, and lidocaine for pain. He was discharged with amoxicillin/clavulanic acid and Ciprodex ear drops with return precautions.

**Figure 1:**
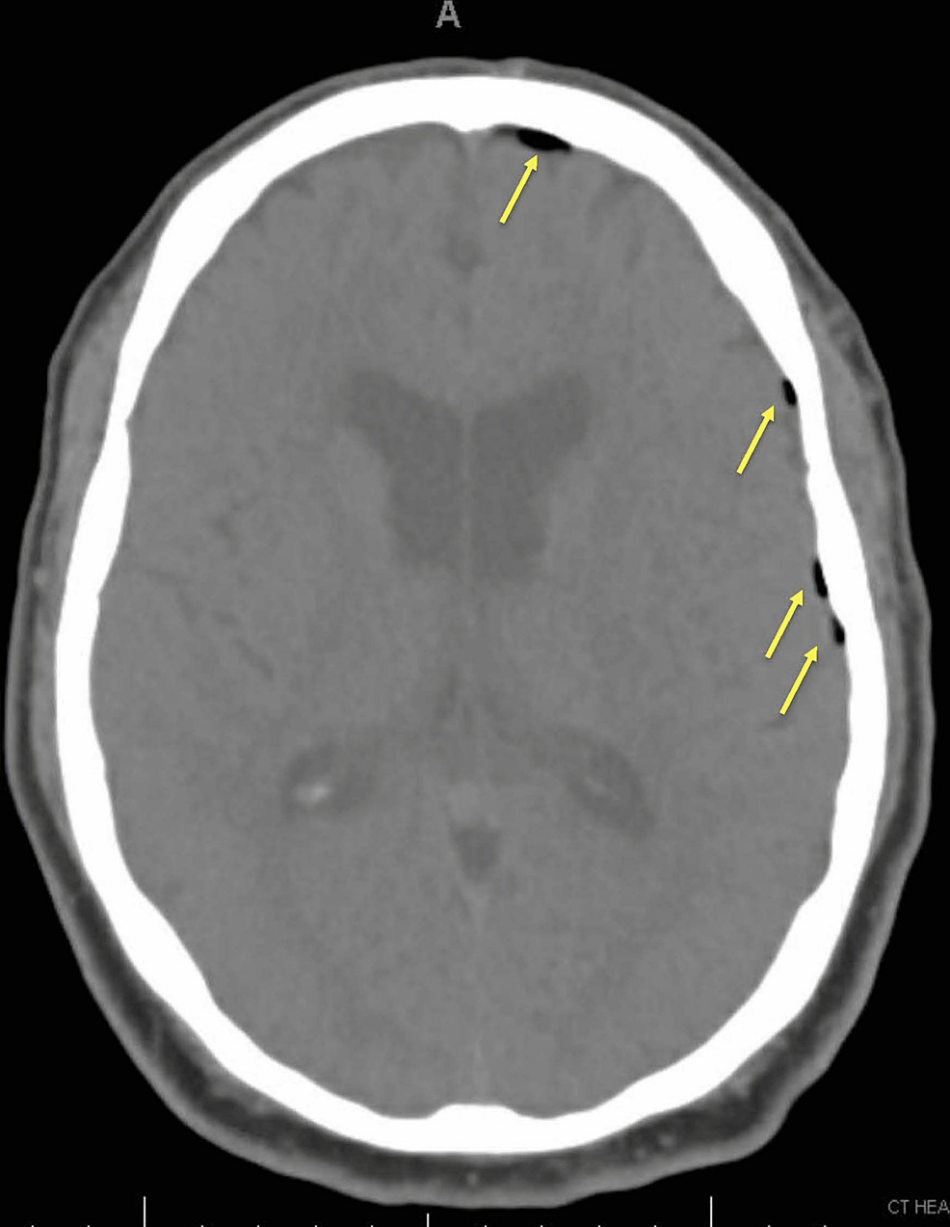
CT of the head with and without contrast showed scattered left frontotemporal pneumocephalus along the dural margins (yellow arrows).

An hour later while at home, the patient developed a worsening left-sided headache, difficulty breathing, chest discomfort, and vague abdominal pain provoking him to present to the emergency department. He was noted to be febrile to 101.3°F and newly toxic appearing. An electrocardiogram (EKG) was only significant for sinus tachycardia. An infectious workup showed leukocytosis with a white blood cell count of 18.9 K/uL, and blood and urine cultures were obtained. He continued to experience a neurologic decline with worsening headache and confusion, prompting a head CT scan, which showed frontotemporal pneumocephalus along the dural margins as shown in Figure 1. He was placed on a 100% fraction of inspired oxygen (FiO2), heated high-flow nasal cannula, and transferred to a high acuity facility to the neurocritical care unit.

**Figure 2:**
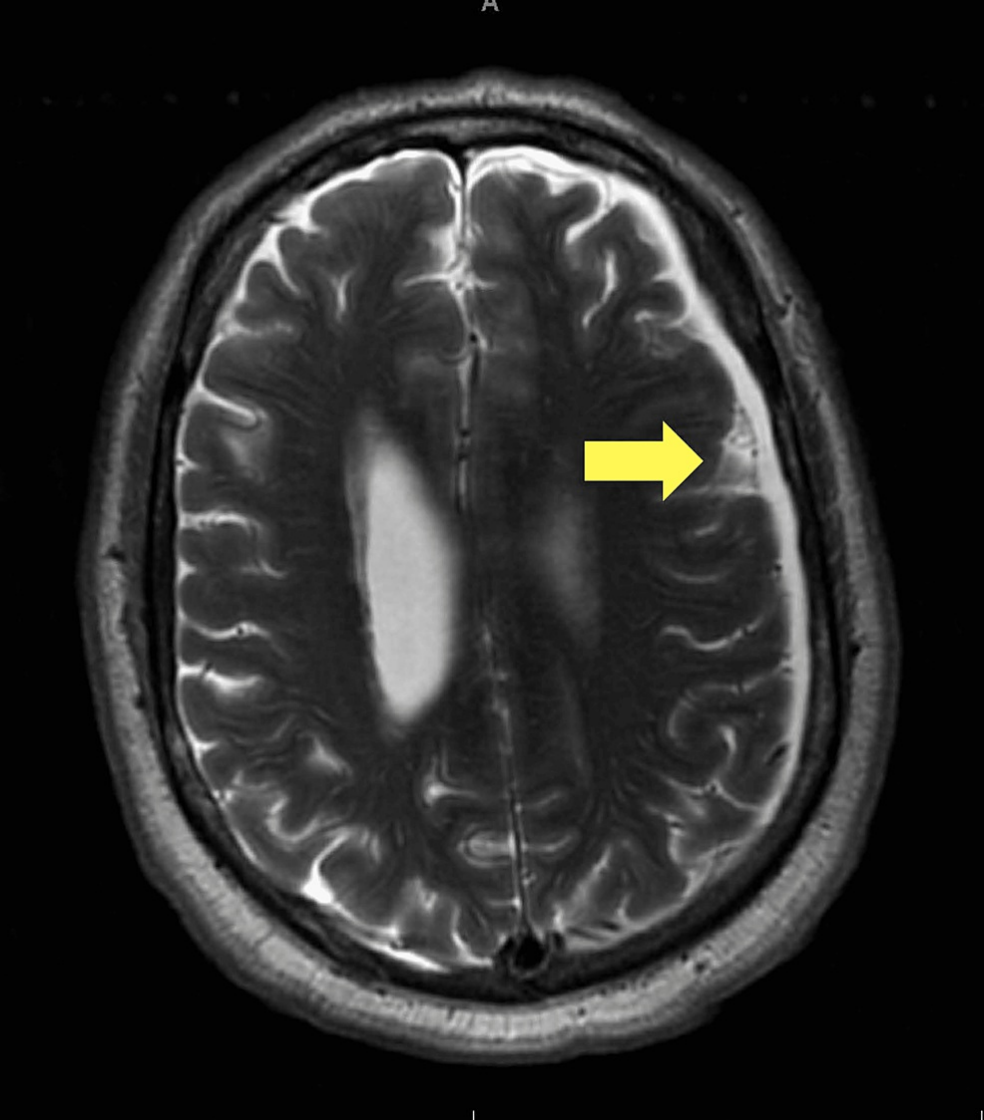
MRI with and without contrast showed subdural fluid collection over the left cerebral convexity suggestive of subacute/chronic subdural hemorrhage versus infectious process.

An otolaryngologist (ENT) evaluated the patient and noted ruptured tympanic membranes. An infectious disease specialist was consulted, given the associated pneumocephalus findings, and their service recommended a regimen of IV ceftriaxone, vancomycin, and metronidazole for suspected otogenic pneumocephalus and he continued to receive Ciprodex ear drops. Blood cultures resulted positive for group A Streptococcus (GAS) on two of the two samples. Vancomycin was discontinued. An EKG showed new-onset atrial fibrillation (AF) with rapid ventricular response. Amiodarone was initiated with a loading dose, and metoprolol and heparin drips were added. Magnetic resonance imaging with and without contrast and venous flow was done under anesthesia with intubation, given the patient’s excruciating discomfort and agitation. Results are as shown in Figure 2, i.e., a 6 mm subdural empyema adjacent to the pneumocephalus. He remained intubated overnight due to agitation and hallucinations. It was verified that he did not have a history of alcohol or substance abuse. A lumbar puncture was done the following day, which revealed increased opening pressure, leukocytes without bacteria, and negative meningitis testing. Table 1 shows consistency with bacterial meningitis. The patient was extubated thereafter.

**Table 1:** Results of lumbar puncture of 10 cc CSF. Definitions: Ab, antibody; DNA, deoxyribonucleic acid; HHV, human herpes virus; HSV, human simplex virus; Ig, immunoglobulin; PCR, polymerase chain reaction; RNA, ribonucleic acid.

Lab test	Lab result	Reference range (units)
Glucose	71	40-70 mg/dl
Protein	94.6	15-45 mg/dl
Cell count		
Nucleated cells	1203	(0-5 # /uL)
Red blood cells		
Neutrophils	90	Rel %
Lymphocytes	6	Rel %
Monocytes	4	Rel %
Meningitis PCR		
Escherichia coli K1	Not detected	Not detected
Haemophilus influenzae	Not detected	Not detected
Listeria	Not detected	Not detected
Neisseria meningitidis	Not detected	Not detected
Streptococcus agalactiae	Not detected	Not detected
Streptococcus pneumoniae	Not detected	Not detected
Cytomegalovirus	Not detected	Not detected
Enterovirus RNA	Not detected	Not detected
HSV 1/2	Not detected	Not detected
HHV6	Not detected	Not detected
Human parechovirus	Not detected	Not detected
Varicella zoster DNA	Not detected	Not detected
Cryptococcus neoformans	Not detected	Not detected
West Nile virus PCR		
IgG	<1.30	<1.30, Ab not detected; 1.30-1.49, equivocal; >1.50 Ab detected
IgM	<0.90	<0.90, Ab not detected; 0.90-1.10, equivocal; >1.10 Ab detected
Opening pressure	36	5-20 mm Hg, normal; normal or mildly increased, viral; >30, bacterial; N/A fungal
Closing pressure	26	5-20 mm Hg

**Figure 3:**
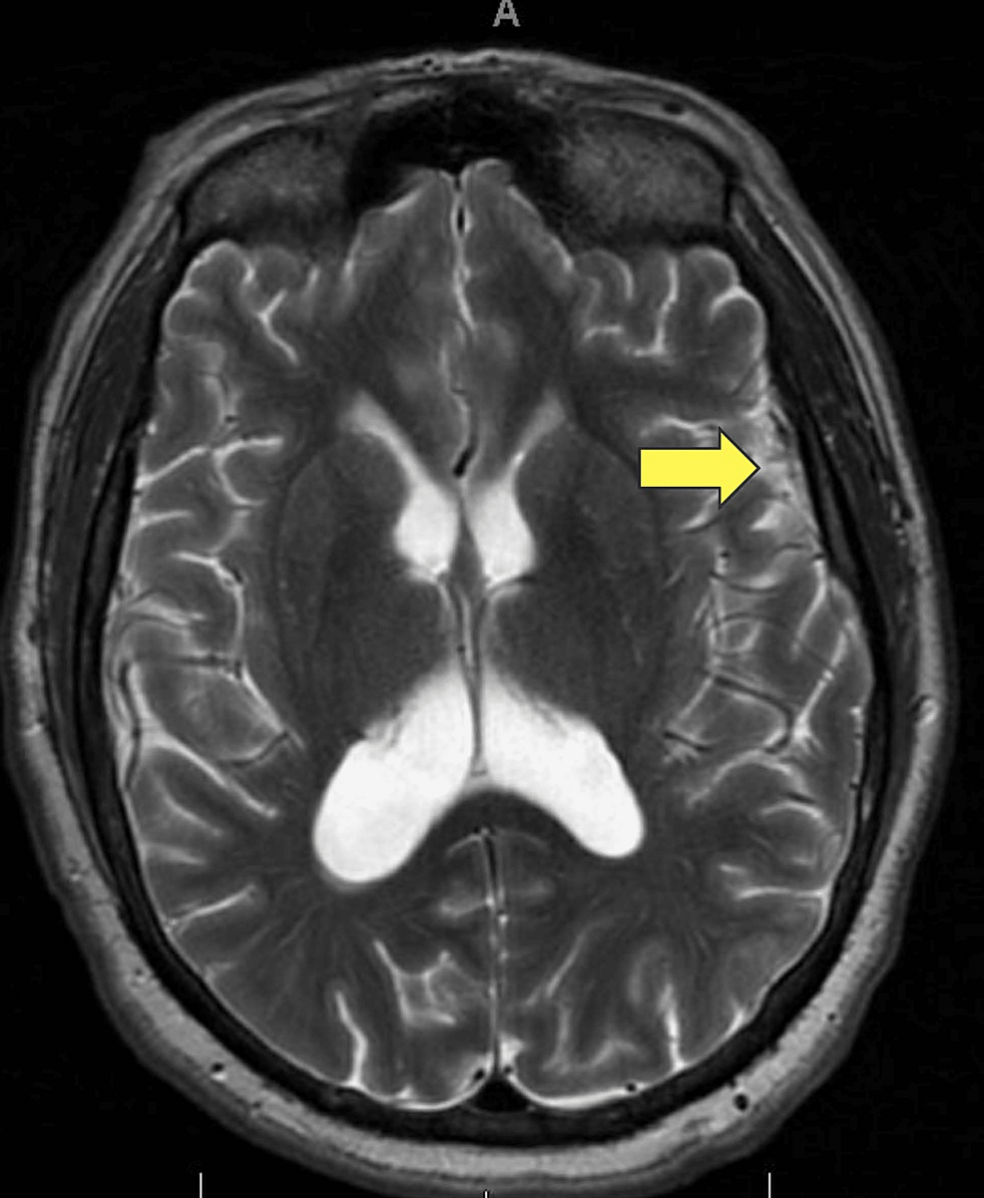
Brain MRI-seizure protocol without and with gadolinium showed an interval decrease of the left frontal region, which previously measured 6 mm in thickness, now measuring 4 mm.

Over the course of his 13-day hospitalization, he started to show improvement a week from admission with treatment. Leukocytosis was downtrending on serum labs, and repeat blood cultures showed no growth. His arrhythmia persisted but was able to be rate controlled. Symptomatically, he continued to feel clogged in his ears, but his headaches improved, and the visual hallucinations dissipated. By the 10th day of his hospitalization, he was at ease, comfortably seated, and had no issues with his oral intake. He still experienced lightheadedness and dizziness with standing and ambulation, but this was somewhat baseline for him despite a negative history of stroke. Repeat MRI showed decreased fluid collection from 6 mm to 4 mm, though there was still extensive fluid throughout the middle ear and mastoid air cells (Figure 3).

**Figure 4:**
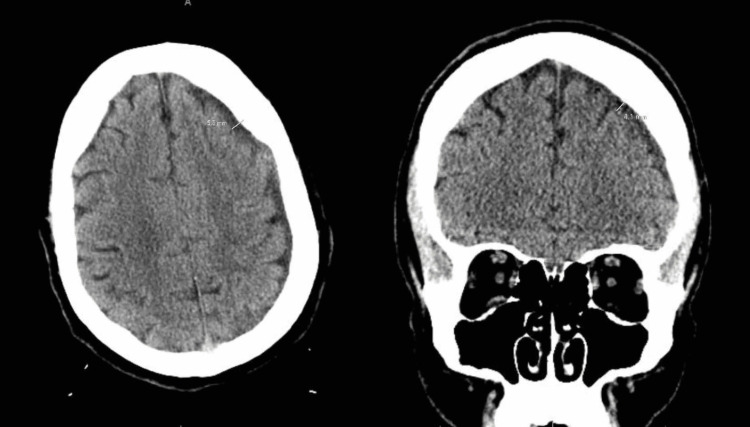
CT of the head without contrast showing changes from January to the follow-up in March. The interval decreased from 5.6 mm to 4.1 mm.

The final CT showed no persistent pneumocephalus (Figure 4). A peripherally inserted central catheter (PICC) was placed with a plan for continued antibiotic therapy with six weeks of IV ceftriaxone and oral metronidazole. Other new medications at discharge included amiodarone, an increased dose of metoprolol, and apixaban. He was discharged with the endorsement of his stable general health status, accompanied by home healthcare assistance.

At a two-week follow-up with primary care, the patient was well appearing. He still complained of a cloudy sensation in his ears and was given decongestants. After completion of his antibiotic course, he did not experience recurrence.

## Discussion

Pneumocephalus is a rare finding, especially secondary to infection. For 295 cases of pneumocephalus, trauma (73.9%) was the leading cause and infection was third (8.8%). The gold standard for the diagnosis of traumatic pneumocephalus is CT, which can detect 0.5 mL of air compared to 2 mL that must be present to be detectable on a standard X-ray [8].

For traumatic pneumocephalus, prophylactic antibiotics are not recommended, even though rates of complication with meningitis in such patients are around 20% [8]. Spontaneous, non-traumatic pneumocephalus should always raise the suspicion of meningitis, and our case was well done with early detection and initiation of CNS penetrable antibiotics [9]. The opening pressure on the lumbar puncture, polymorphonuclear prominence, and negative meningitis PCR indicated bacterial meningitis related to pneumocephalus from AOM. He was empirically treated according to blood culture results of GAS.

While complications of AOM are rare (0.5-4%), mortality ranges from 5% to 15%. Pneumocephalus is not the only such complication but is one of the more fatal ones. Poor dentition and diabetes mellitus were identified risk factors in our patient, placing him at increased risk for CNS infection; however, there are little data about dependent risk factors for infectious pneumocephalus [10].

The causative organism, GAS, is of interest. Current literature on pneumocephalus secondary to infection focuses heavily on *S. pneumoniae*, with GAS being a rare and dangerous finding. CDC data from 2005 to 2012 showed a case-fatality rate of 11.7% for invasive GAS infections [11]. Appropriate antibiotic therapy is essential in such cases, and an important consideration aside from organism sensitivities, in this case, is CNS penetration. While both fluoroquinolones and metronidazole exhibit significant penetration into the CSF, it is important to take into account the elevated resistance rates associated with fluoroquinolones. A more traditional picture of* S. pneumoniae* infection would respond well to cephalosporins and vancomycin [12]. Thus, the patient received both adequate prophylactic and therapeutic antibiotics. Medical management is the standard for infectious pneumocephalus cases, as they tend to resolve with the resolution of the infection. Surgical interventions are quite invasive, but if seizures or other signs of severe neurologic deficit had developed, surgical decompression would have been indicated [6].

Finally, our patient’s course was complicated by AF, which is a common complication of sepsis and severe infections. In fact, sepsis is associated with a six-fold increased risk of developing arrhythmia in the first three days of hospitalization. Anticoagulation is generally indicated in the acute setting of AF, but no consensus has been reached on the benefits of long-term anticoagulation [13]. This provides an interesting dilemma in a patient with pneumocephalus and AF, as the risk of hemorrhage is high and must be weighed against the risk of a mural thrombus leading to ischemic stroke. Ultimately, our patient was stabilizing, so a heparin drip was started for stroke and deep venous thrombosis prophylaxis, but more aggressive measures were not taken. Such patients should be evaluated on a case-by-case basis with patient involvement in decision-making if able, but again more statistics would make this process much easier.

## Conclusions

Pneumocephalus is a rare and complicated disease with a variety of etiologies. In this case study, we present a patient with pneumocephalus, subdural empyema, and GAS growth secondary to AOM. His course was complicated by arrhythmia. Our patient was treated with a standard six-week course of antibiotics consisting of IV ceftriaxone and oral metronidazole, which have been shown to have essential CNS penetration. He ultimately showed improvement and has not had a recurrence of the infection. While the literature on pneumocephalus focuses on traumatic etiologies, given they compose the majority of cases, infectious causes are an important area of study, as they pose their own unique risks and considerations. Additionally, there is not sufficient research on predisposing risk factors. In a patient with a background history of diabetes mellitus, poor dentition, and obesity, which each generally increases the risk of illness, there are no data about specific risk factors for infectious pneumocephalus. While it is a difficult task, the rate of infectious pneumocephalus cases in the US is fortunately low; however, future research should distinguish the causes of AOM and secondary meningitis. It is also encouraged to ascertain potential dependent risk factors to help us better recognize, understand, and treat such conditions.
